# Expression of *secretory calcium-binding phosphoprotein* (*scpp*) genes in medaka during the formation and replacement of pharyngeal teeth

**DOI:** 10.1186/s12903-023-03498-7

**Published:** 2023-10-11

**Authors:** Tsuyoshi Morita, Shin Matsumoto, Otto Baba

**Affiliations:** 1 Department of Oral and Maxillofacial Anatomy, Institute of Biomedical Sciences, Tokushima University Graduate School, 3-18-15, Kuramoto-cho, Tokushima-shi, Tokushima, 770-8504 Japan; 2 Oral Surgery Department, St. Luke’s International Hospital, 9-1, Akashi-cho, Chuo-ku, Tokyo, 104-8560 Japan

**Keywords:** *Scpp*, Medaka, Pharyngeal teeth, In situ hybridization

## Abstract

**Background:**

Analyses of tooth families and tooth-forming units in medaka with regard to tooth replacement cycles and the localization of odontogenic stem cell niches in the pharyngeal dentition clearly indicate that continuous tooth replacement is maintained. The *secretory calcium-binding phosphoprotein* (*scpp*) gene cluster is involved in the formation of mineralized tissues, such as dental and bone tissues, and the genes encoding multiple SCPPs are conserved in fish, amphibians, reptiles, and mammals. In the present study, we examined the expression patterns of several *scpp* genes in the pharyngeal teeth of medaka to elucidate their roles during tooth formation and replacement.

**Methods:**

Himedaka (Japanese medaka, *Oryzias latipes*) of both sexes (body length: 28 to 33 mm) were used in this study. Real-time quantitative reverse transcription-polymerase chain reaction (PCR) (qPCR) data were evaluated using one-way analysis of variance for multi-group comparisons, and the significance of differences was determined by Tukey’s comparison test. The expression of *scpp* genes was examined using in situ hybridization (ISH) with a digoxigenin-labeled, single-stranded antisense probe.

**Results:**

qPCR results showed that several *scpp* genes were strongly expressed in pharyngeal tissues. ISH analysis revealed specific expression of *scpp1*, *scpp5*, and *sparc* in tooth germ, and *scpp5* was continually expressed in the odontoblasts of teeth attached to pedicles, but not in the osteoblasts of pedicles. In addition, many *scpp* genes were expressed in inner dental epithelium (ide), but not in odontoblasts, and *scpp2* consistently showed epithelial-specific expression in the functional teeth. Taken together, these data indicate that specific expression of *scpp2* and *scpp5* may play a critical role in pharyngeal tooth formation in medaka.

**Conclusion:**

We characterized changes in the expression patterns of *scpp* genes in medaka during the formation and replacement of pharyngeal teeth.

**Supplementary Information:**

The online version contains supplementary material available at 10.1186/s12903-023-03498-7.

## Background

Medaka (*Oryzias latipes*) is a small, egg-laying freshwater fish [1] that has a smaller genome size than zebrafish [2]. Medaka have oral and pharyngeal dentition, and adult fish have more than a thousand teeth [3]. The pharyngeal teeth of medaka are a conical in shape and are connected to the pharyngeal bone via a tooth-supporting bone called the pedicle or attachment bone [4, 5]. The functional teeth are attached to the pedicle either by ankylosis in the lower teeth or by fibrous connections in the upper teeth [6]. A previous study identified tooth families and tooth-forming units in medaka and determined the tooth replacement cycles and the localization of odontogenic stem cell niches in the pharyngeal dentition, providing insights into these mechanisms and clearly indicating that continuous tooth replacement is maintained [4]. Furthermore, tooth replacement in medaka occurs due to the turnover of pedicles by osteoblasts and osteoclasts [5].

The *secretory calcium-binding phosphoprotein* (*scpp*) gene cluster functions in mineralized tissues such as dental and bone tissues, and the genes encoding multiple SCPPs are reportedly conserved in fishes, amphibians, reptiles, and mammals [7–9]. SCPPs can be classified into two subclasses: acidic SCPPs and Pro/Gln (P/Q)-rich SCPPs [8]. Acidic SCPPs function in bone, dentin, and cementum formation, whereas P/Q-rich SCPPs are involved in enamel and enameloid formation [8–10]. Since enameloid is derived partly or mainly from mesenchymal cells, it differs from enamel formation. In enameloid, odontoblasts provide an organic matrix containing collagen that is then dissolved and degenerated by epithelial cells, which supply inorganic ions during advanced crystal growth [11]. SPP1, odontogenic ameloblast-associated protein (ODAM), and their common ancestor, secreted protein, acidic, cysteine-rich like 1 (SPARCL1), are conserved in jawed vertebrates [12]. In teleost fishes such as fugu and zebrafish, *sparcl1* and other *scpp* genes (except for *spp1*) constitute the *sparcl1*-*scpp* gene cluster [12]. The *sparcl1*-*scpp* gene cluster of zebrafish and fugu is located on chromosome 1 and chromosome 17, respectively, whereas in medaka, it is also located on chromosome 1. Other members of the *scpp* gene cluster are likely more specific between lineages, indicating that they arose with lineage-specific duplications and deletions, which possibly resulted in the specialization of certain *scpp* family genes in vertebrates [12, 13]. A recent study showed that the formation of ganoid scales in ray-finned fish involves the largest known repertoire of *scpp* genes, which includes many genes previously unknown in teleosts [13, 14]. In addition, that study confirmed that *scpp6* and *fa93e10* are teleost orthologues of *ameloblastin* (*ambn*) and *enamelin* (*enam*), respectively [13].

Previous studies characterized the expression patterns of *scpp* genes during the tooth germ stage of fugu [7], zebrafish [8, 15], and cichlid [16]. In the tooth germ stage of fugu, a previous study reported that the expression patterns of *scpp* genes, *scpp2*, *scpp4*, and *scpp5* in the ide, *scpp1*, and *scpp5* in odontoblasts, and *scpp3a* and *scpp3b* in the ide [7]. The expression patterns of *scpp1*, *scpp2*, and *scpp5* in zebrafish were similar to those of fugu. In addition, *scpp*9 was strongly expressed in the ide, and *spp1* was strongly expressed in the cells of the pedicle and jawbone [8]. In zebrafish, expression of ambn and enam was detected in ide cells [15]. *Scpp2* and *scpp5* in the cichlid were also similar to the expression patterns of fugu and zebrafish [16]. Recently, it was reported that *scpp5* in the pharyngeal tissues of zebrafish was expressed in the odontoblasts, but not pedicles (dentinous bone) [17]. In the present study, we examined the expression patterns of several *scpp* genes in the pharyngeal teeth of medaka to elucidate their roles during tooth formation and replacement.

## Methods

### Medaka

Himedaka (Japanese medaka, *Oryzias latipes*) of both sexes (*n* = 22, body length: 28 to 33 mm) were used in this study. The fish were maintained in the laboratory at a water temperature of 23–25 °C under a 14-h/10-h light/dark cycle. All animal experiments were performed in accordance with policies and protocols approved by the guidelines of the Animal Welfare Committee of Tokushima University. In addition, we confirmed that all of the methods are in accordance with ARRIVE guidelines 2.0 for reporting of animal experiments.

### Cloning of *scpp* genes in medaka

Genome Data Viewer (GDV) (https://www.ncbi.nlm.nih.gov/genome/gdv/) [18] and Ensembl Genome Browser (EGB) 105 (https://www.ensembl.org/index.html) were used to search gene sequence databases to identify gene orthologues and examine the expression of *scpp* genes in medaka (ASM223467v1). Genomic synteny analysis was also conducted using GDV and EGB105. Each *scpp* gene assembly number in medaka was referenced to GDV and EGB105, as listed in availability of data. For *scpp4*, *scpp7*, *scpp9*, and *enam*, similar sequences were identified in the medaka genome by performing genomic alignment with zebrafish (GRCz11) and fugu (fTakRub1.2) in EGB105. Those sequences were then obtained by searches using BLAST (blastin) (https://blast.ncbi.nlm.nih.gov/Blast.cgi). Genomic locations of the *scpp* genes were extracted from publicly available annotations in EGB105. Specific primers were designed using Primer3Plus (https://primer3plus.com), and the primer sequences are listed in Table 1. Brain, liver, fin ray bone, and pharyngeal tissues (teeth and bone) were dissected from the medaka, and total RNA was extracted from each tissue using TRI REAGENT (Molecular Research Center Inc., Cincinnati, OH, USA). Total RNA from the fin ray bone was extracted after dissecting the fin ray from the body without removing the epithelium. cDNAs were generated using a 1st Strand cDNA Synthesis kit for RT-PCR® (Roche, Basel, Switzerland).

**Table 1:** Specific primers for *scpp* genes and mineralized tissue-related genes used in qPCR and ISH analyses

Gene	Primer sequences (5' → 3')	Size	Primer sequences (5' → 3')	Size
	qPCR		ISH	
*scpp1*	F: GCCAGCAAGAGTTCAAAGGA	139 bp	F: CAGACAACATCGTGAGACTTCTTC	396 bp
	R: CATCTCCTACGCTCTGGACAA		R: GAAAACACCCTTACCCAACTCTG	
*scpp2*	F: TGCCAGCAATAGCAATGAGA	148 bp	F: CCTTCTGTTGATCTGCCTTTTC	340 bp
	R: CTGGGGGTTTAGATTCAGCA		R: CTGAGGGATGTTGGGATTATTG	
*scpp5*	F: CATGCCTCAGCAAACCATAAA	149 bp	F: GTGTTTTGTTTCTGAGGGGACA	443 bp
	R: GGCAGATAATGACGGAGGAG		R: GTTTGCTGAGGCATGTTTTGAG	
*sparc*	F: TGAGGGACTGGCTGAAGAAC	150 bp	F: AGCAGAAGCTCAGGGTAAAGAAG	352 bp
	R: CCAACAGGTCCAAAGAGTGG		R: GTCAGAGGAGTTTAGATGACGAGA	
*spp1*	F: CACTGACTTTCTGGAGGAGGA	84 bp	F: AAGAAGACGAAGACGAAACCAC	252 bp
	R: TGGTCTTGAGATACGCTGGA		R: AGGGAGAGGGAACTTTGTGATAG	
*col1a1*	F: CAAGAACAGCGTTGCCTACA	95 bp	F: TGAAGTGGTGTGTGAAGAAGTG	284 bp
	R: CCTCGGCTCTGATCTCAATC		R: GCATAGCTGGAGATTTGTCATC	
*bglap*	F: TGAAATGGCTGACACTGAGG	52 bp	F: CTACTGCTTCTGCCTCATCATC	215 bp
	R: TCCGTAGTAGGCGGTGTATG		R: AGTGTCAGCCATTTCATCACAG	
*runx2*	F: GGAAAGGATGCGAGTGAGAG	105 bp	F: GCACACCCTATCTCTACTACGG	265 bp
	R: TCTGTGATCTGCGTCTGACC		R: CTCCACCCACTTTTCCTCTAA	
*sp7*	F: GCTCACGGTTTAAGGAGGTG	73 bp	F: CGCATCTATTCTGGAGGTAGGAAG	351 bp
	R: AATCAGGGATGGAGGGAAAC		R: GAGTGGGAGAAGGGGTTATATTCTG	
*β-actin*	F: GCCAACAGGGAGAAGATGAC	133 bp		
	R: CATCACCAGAGTCCATGACG			

### Real-time quantitative reverse transcription-polymerase chain reaction (PCR) (qPCR)

qPCR was performed using PowerUp™ SYBR® Green Master Mix (Applied Biosystems™, Thermo Fisher Scientific k.k., Tokyo, Japan) and a MyGo mini system (IT-IS Life Science Ltd., Dublin, Ireland). The qPCR conditions for amplification were as follows: initial denaturation at 95 °C for 120 s, followed by 40 cycles of 10 s at 95 °C and 30 s at 60 °C. The length of each amplicon was approximately 50–150 base pairs, and the confirmed efficacy of each specific primer pair was 2.0. Gene expression levels were normalized to those of *β-actin*. The expression levels in each sample were compared to those from the fin ray bone (at level 1). Three different samples were analyzed, and reactions were repeated three times. Relative mRNA expression levels were calculated by the ΔΔCt method. All data were calculated as the mean ± standard deviation (SD). Data were analyzed using one-way analysis of variance for multi-group comparisons, and the significance was determined by Tukey’s comparison test using SPSS Statistics software (version 24, IBM Inc., Chicago, IL, USA). A *p*-value of < 0.05 or < 0.01 was considered statistically significant.

### Histological analysis

The heads of medaka were fixed in a 4% paraformaldehyde solution (0.1 M phosphate buffer, pH 7.4) at 4 °C overnight, decalcified in 8% ethylenediaminetetraacetic acid (EDTA) solution at 4 °C for 3 days, then embedded in paraffin according to conventional methods. The prepared paraffin blocks were cut into sagittal and horizontal sections at a thickness of 5 µm.

Pharyngeal tissues of medaka were fixed in 4% paraformaldehyde solution (0.1 M phosphate buffer, pH 7.4) at 4 °C overnight, and then the samples were embedded in Technovit 8100 (Heraeus Kulzer, Wehrheim, Germany) with a coagulant (Heraeus Kulzer). The resin blocks were cut into horizontal sections at a thickness of 1 µm. Toluidine blue staining and tartrate-resistant acid phosphatase (TRAP) reaction were performed to identify osteoclasts [3].

### In situ hybridization (ISH)

ISH was performed as described previously [19]. Briefly, template cDNAs for mRNA probes were generated using gene-specific primer sets encoding T3 or T7 RNA polymerase promoter sequences. The primer sequences are listed in Table 1. The RT-PCR products for ISH were generated using Ampdirect® Plus (Shimazu, Japan) with a Mastercycler® nexus gradient (Eppendorf, Japan). The PCR conditions were as follows: initial denaturation at 94 °C for 120 s, followed by 35 cycles of 30 s at 94 °C, 30 s at 60 °C, and 60 s at 72 °C. Digoxigenin (DIG)-labeled, single-stranded antisense and sense strands were then generated from the PCR products using a DIG RNA Labeling kit® (Roche). After de-waxing, histological sections were treated in the following solutions: Proteinase K (1.0 µg/ml, Sigma) in 10 mM Tris–HCl buffer (pH 8.0) containing 1.0 mM EDTA at 37 °C for 10 min; 0.2 M HCl at 25 °C for 10 min; and 0.1 M triethanolamine containing 0.5% acetic anhydride at 25 °C for 15 min for acetylation. Hybridization was performed by incubating the samples with diluted RNA probes (1.0 µg/ml) at 50–60 °C overnight. The sections were washed according to conventional protocols. Samples were visualized using an anti-DIG antibody as follows: sections were incubated with 1.5% blocking buffer at 25 °C for 1 h and then incubated with alkaline phosphatase–labeled anti-DIG antibody (Roche) at 25 °C for 2 h. Finally, the sections were reacted with BCIP/NBT (Roche) solutions and developed at 25 °C overnight. The sections were counterstained with methyl green.

## Results

### Synteny analysis of *scpp* in medaka

**Fig. 1:**
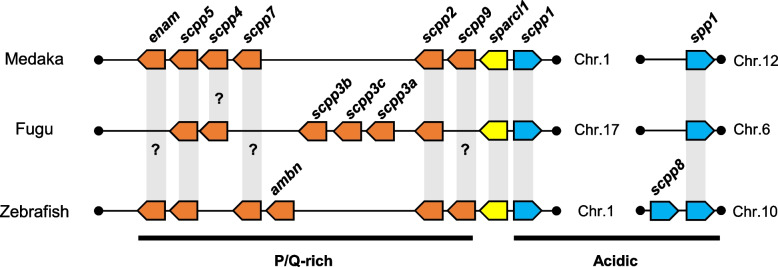
Synteny analysis of *secretory calcium-binding phosphoprotein* (*scpp*) in medaka, fugu, and zebrafish. Schematic illustration of the location and transcriptional orientation of the *sparcl1* (yellow box), Pro/Gln-rich *scpp* (orange box), and acidic *scpp* (blue box) genes. Teleost orthologues (gray vertical bars) obtained from GDV and EGB105 are shown (orthologous were). In addition, further putative orthologues supported by low sequence similarities (?) connect *scpp4*, *scpp7*, *scpp9*, and *enam*, respectively. ambn: ameloblastin; enam: enamelin; Chr: chromosome

The GDV was used to search for orthologues of medaka *scpp1*, *scpp2*, *scpp5*, and *sparc* in zebrafish (GRCz11) and fugu (fTakRub1.2). For *scpp4*, *scpp7*, *scpp9*, and *enam*, similar sequences were identified in the medaka genome by performing genomic alignment with zebrafish and fugu using EGB105. The resulting sequences were then searched for using BLAST (blastin). Genomic locations of the *scpp* genes were extracted from publicly available annotations in EGB105. The location and transcriptional orientation of the *scpp* genes are schematically illustrated in Fig. 1. Of the various *scpp* genes, expression of *scpp3* or *ambn* orthologues was not observed in the present study. However, genomic alignment analysis showed that fugu *scpp3a* and *scpp3b* are similar to a non-coding RNA in medaka. Fugu *scpp3a* was similar to uncharacterized LOC101161295 ncRNA or uncharacterized LOC110015140 ncRNA, whereas fugu *scpp3b* was similar to uncharacterized LOC101160534 ncRNA or uncharacterized LOC101161034 ncRNA. As these are non-coding RNAs, they were not considered in the present study. Fugu *scpp3c* did not show sequence similarity to that gene in medaka.

### Expression levels of *scpp* and mineralized tissue-related genes in medaka

**Fig. 2:**
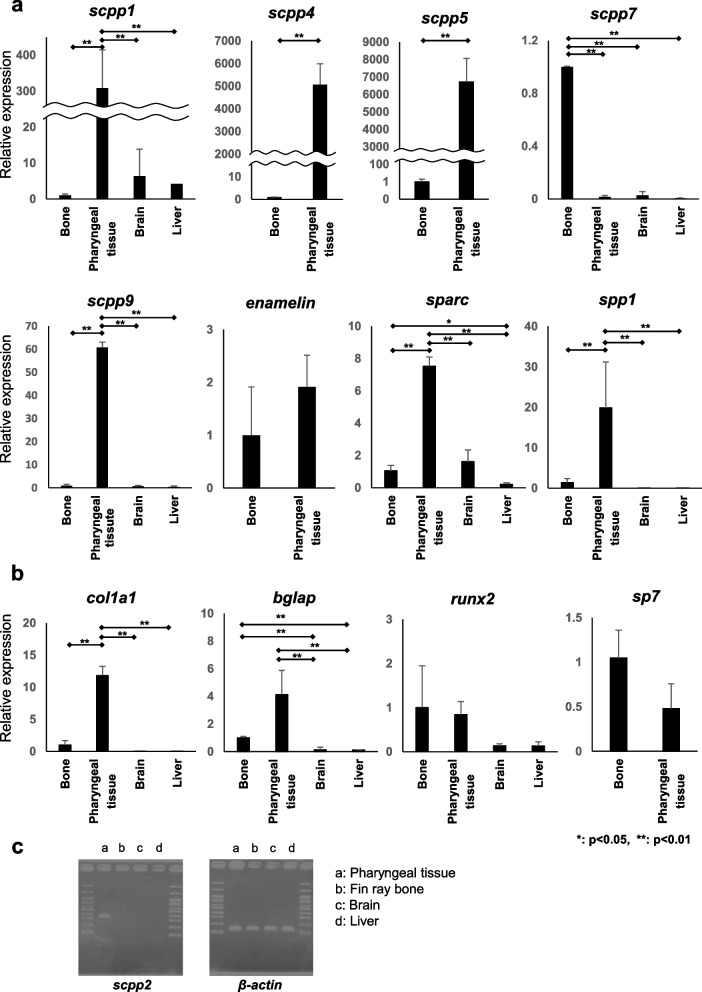
Results of qPCR indicating expression levels of *scpps* and mineralized tissue-related genes in medaka. The qPCR results are obtained from an experiment in which the expression levels of target genes and an endogenous control (*β-actin*) are evaluated. The expression levels in each sample were compared to those in fin ray bone. **a** Transcripts for *scpp1*, *scpp9*, *sparc*, and *spp1* were detected in the pharyngeal tissues, fin ray bone, brain, and liver. Transcripts for *scpp4*, *scpp5*, and *enam* were detected in the mineralized tissues but not in the soft tissues, and the expression levels of *scpp4* and *scpp5* were significantly higher in the pharyngeal tissues than in fin ray bone. Transcripts for *scpp7* were detected in each tissue, and the expression levels were significantly higher in fin ray bone than in other tissues. **b** Transcripts for *collagen 1a1* (*col1a1*) and *bone gla protein* (*bglap*) were detected at higher levels in mineralized tissues than in other tissues. Transcripts for *runt-related transcription factor 2* (*runx2*) were found to be expressed at higher levels in mineralized tissues than in soft tissues. Transcripts for *sp7* were only detected in mineralized tissues, and no significant difference in expression level was found between the pharyngeal tissues and fin ray bone. **c** Results of PCR analysis of the expression of *scpp2* in medaka. Gene expression levels were normalized to those of *β-actin*. Data were presented as the mean ± SD (*n* = 3). *p < 0.05, **p < 0.01

The expression levels of transcripts for *scpp1*, *scpp9*, *sparc*, and *spp1* were analyzed in the pharyngeal tissues, fin ray bone, brain, and liver (Fig. 2a). The expression levels of these genes were significantly higher in the pharyngeal tissues than in other tissues (Fig. 2a). Transcripts for *scpp2* were detected only in the pharyngeal tissues (Fig. 2c). Transcripts for *scpp4*, *scpp5*, and *enam* were detected in mineralized tissues, but not in soft tissues, and the expression levels of the *scpp4* and *scpp5* transcripts were significantly higher in pharyngeal tissues than in fin ray bones (Fig. 2a). However, *enam* expression levels did not differ significantly between the mineralized tissues, such as bone and teeth (Fig. 2a). Transcripts for *scpp7* were detected in each tissue, and the expression levels were significantly higher in fin ray bone than in other tissues (Fig. 2a). Levels of transcripts for *collagen 1a1* (*col1a1*) and *bone gla protein* (*bglap*) were higher in mineralized tissues than in other tissues (Fig. 2b). The levels of transcripts for *runt-related transcription factor 2* (*runx2*) were higher in mineralized tissues than in soft tissues (Fig. 2b). Transcripts for *sp7* were detected only in mineralized tissues, and no significant difference in expression level was found between the pharyngeal tissues and fin ray bone (Fig. 2b). In addition, there was no significant difference between the expression levels of transcripts for *runx2* and *sp7* in the tissues examined.

### Expression patterns of *scpp* and mineralized tissue-related genes during tooth germ stages

**Fig. 3:**
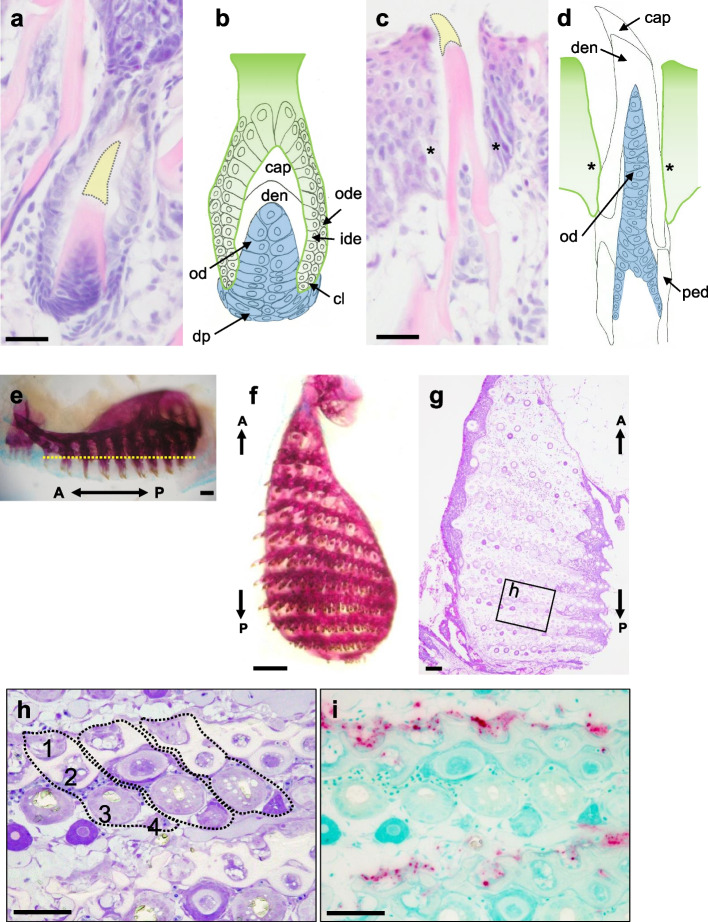
Histological image of tooth families in the pharyngeal tissues. **a**, **c** Hematoxylin–eosin staining of upper tooth germ and the functional tooth in the pharyngeal tissues. **b**, **d** Schematic diagram of tooth germ and the functional tooth. **e**, **f** Alizarin red staining of the upper left of pharyngeal tissue. **g** Toluidine blue staining of the pharyngeal tissue was cut at the level of the pedicles under the erupted functional teeth for Fig. 3e (yellow dotted line). **h** High-magnification views of the boxed areas in (**g**), showing two pedicles and two tooth germs in a row. These structures were identified as the first pedicle (1), second pedicle (2), third tooth germ (3), and fourth tooth germ (4) based on their developmental stages. **i** TRAP staining of the horizontal section of the pharyngeal teeth. E: enameloid; D: dentin; ode: outer dental epithelium; ide: inner dental epithelium; dp: dental papilla; ped: pedicle. *: epithelial cells adjacent to the surface of the tooth shaft. A: anterior; P: posterior. Scale bars: 20 µm (**a**, **c**), 200 µm (**e**, **f**), 100 µm (**g**), and 50 µm (**h**, **i**)

In the enameloid organ of the tooth germ, the ide and outer dental epithelium (ode) are clearly distinguishable (Fig. 3a, b). Cap enameloid (acrodin) is shown as the dotted yellow area after decalcification, and dentin was observed adjacent to the cap enameloid. In addition, odontoblasts were observed adjacent to dentin, and dental papilla under the odontoblasts (Fig. 3a, b). Erupted functional teeth were connected to pedicles by collagenous fibers (Fig. 3c, d). In horizontal sections at the height of the pedicles under the erupted teeth (Fig. 3e), two pedicles and two tooth germs were typically observed in a row (Fig. 3 f, g, h, i). In this study, we classified these structures as the first pedicle, second pedicle, third tooth germ, and fourth tooth germ based on their developmental stages (Fig. 3h). Numerous TRAP-positive cells were observed in the anterior side of the first pedicles but not in the second pedicles (Fig. 3h).

**Fig. 4:**
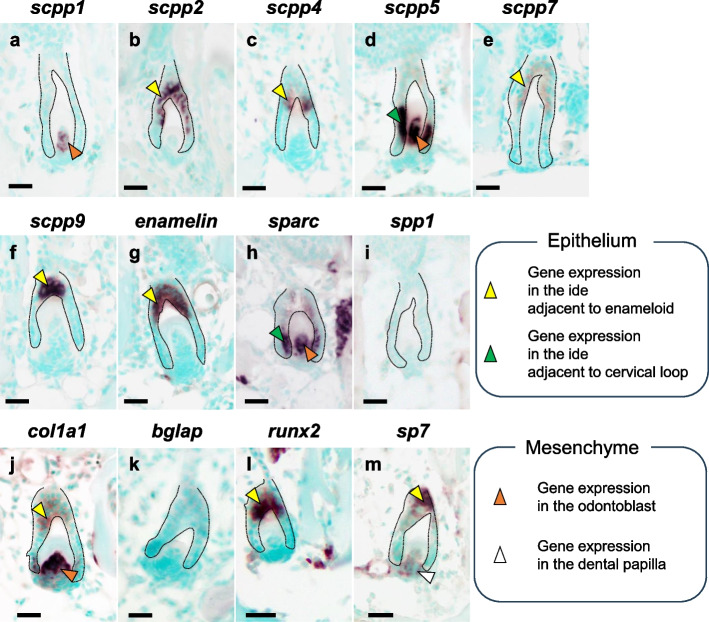
In situ hybridization (ISH) analysis of sagittal sections showing the expression of *scpps* and mineralized tissue-related genes in the upper tooth germs of pharyngeal tissues. Broken line indicates the area of the enameloid organ. Transcripts for *scpp1*, *-5*, *sparc*, *and col1a1* were detected in odontoblasts (orange). Transcripts for *scpp2*, *-4*, *-7*, *-9*, *enam*, *col1a1*, *runx2*, and *sp7* were detected only in the ide adjacent to the enameloid (yellow). Transcripts for *scpp5* and *sparc* were detected in ide adjacent to the cervical loop (green). No *spp1* or *bglap* transcripts were detected in the tooth germ. Transcripts for *sp7* were weakly detected in the dental papilla (white). Scale bars: 20 µm

When tooth germ stages were examined, transcripts for *scpp1* were detected in odontoblasts, but not in the ide and dental papilla (Fig. 4a). Transcripts for *scpp2*, *scpp4*, *scpp9*, and *enam* were detected only in the ide adjacent to enameloid, but not in ide adjacent to the cervical loop (Fig. 4b, c, f, g). Transcripts for *scpp5* and *sparc* were detected in both the ide adjacent to the cervical loop and in odontoblasts during dentin formation, but no transcripts were detected in either the ode or dental papilla (Fig. 4d). Transcripts for *scpp7* were weakly detected in the ide adjacent to enameloid (Fig. 4e). No *spp1* or *bglap* transcripts were detected in the tooth germ (Fig. 4i, k). Transcripts for *col1a1* were weakly detected in the ide adjacent to enameloid and strongly expressed in odontoblasts (Fig. 4j). Transcripts for *runx2* were detected in the ide adjacent to enameloid but not in ide adjacent to the cervical loop or dental papilla (Fig. 4l). Transcripts for *sp7* were weakly detected in the ide adjacent to enameloid and dental papilla, but not in odontoblasts (Fig. 4m).

### Expression patterns of *scpp* and mineralized tissue-related genes in functional teeth

**Fig. 5:**
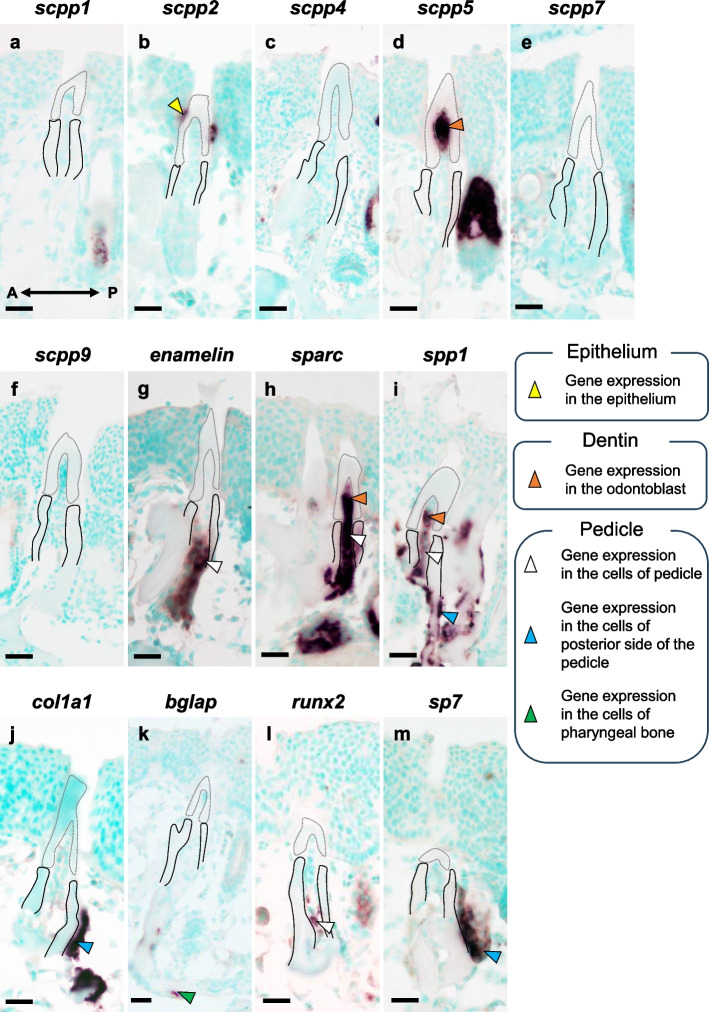
Results of ISH analysis show the expression of *scpps* and mineralized tissue-related genes in the upper functional teeth of pharyngeal tissues. Broken gray line indicates the area of dentin in the pharyngeal teeth. Black line indicates the region of the pedicles. No transcripts for *scpp1*, *-4*, *-7*, and *-9* were detected in the cells of pharyngeal teeth and pedicles. Transcripts for *scpp2* were detected in the epithelial cells adjacent to the surface of the tooth shaft (yellow). Transcripts for *scpp5*, *sparc*, and *spp1* were detected within the odontoblasts (orange). Transcripts for *enam*, *sparc*, and *spp1* were detected within the mesenchymal cells of pedicles (white). Transcripts for *col1a1* and *sp7* were detected in the mesenchymal cells located on the posterior side of the pedicles (blue). Transcripts for *bglap* were detected in osteoblasts of pharyngeal bone (green). Transcripts for *runx2* were detected in mesenchymal cells of the pedicles and weakly detected in the cells surrounding the pedicles (white). A: anterior; P: posterior. Scale bars: 50 µm

**Fig. 6:**
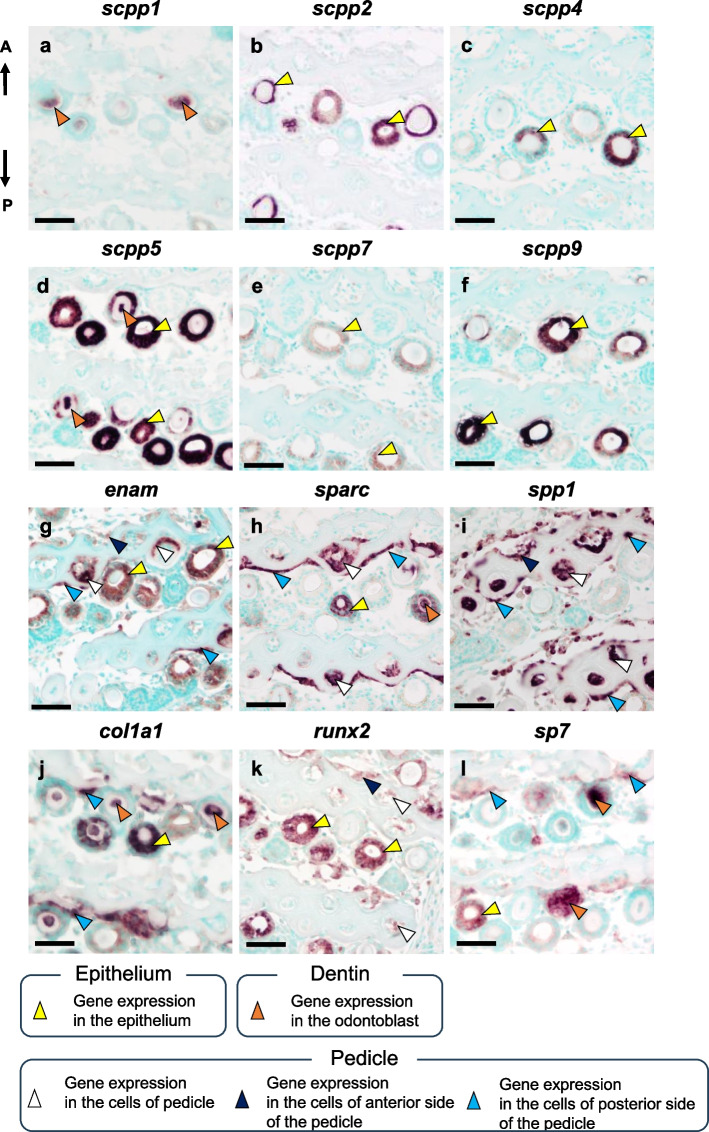
ISH analysis of horizontal sections showing the expression of *scpps* and mineralized tissue-related genes. Transcripts for *scpp1*, *-5*, *sparc*, *col1a1*, and *sp7* were detected in odontoblasts (orange). Transcripts for *scpp2*, *-4*, *-5*, *-7*, *-9*, *enam*, *sparc*, *col1a1*, *runx2*, and *sp7* were detected in the ide (yellow). Transcripts for *enam*, *sparc*, *spp1*, and *runx2* were detected in the mesenchymal cells of pedicles (white). Transcripts for *enam*, *spp1*, and *runx2* were detected in the mesenchymal cells located on the anterior side of the pedicles (dark blue). Transcripts for *enam*, *sparc*, *spp1*, *col1a1*, and *sp7* were detected in the mesenchymal cells located on the posterior side of the pedicles (blue). A: anterior; P: posterior. Scale bars: 50 µm

When functional teeth were examined, no *scpp1*, *scpp4*, *scpp7*, or *scpp9* transcripts were detected in the cells of pharyngeal teeth and pedicles (Fig. 5a, c, e, f). Transcripts for *scpp2* were detected in the epithelial cells adjacent to the surface of the tooth shaft (Fig. 5b). Transcripts for *scpp5* were detected within the odontoblasts above the border between the teeth and pedicles, but not in the cells of the pedicles (Fig. 5d). Transcripts for *enam* were detected in the mesenchymal cells of pedicles, but not in odontoblasts of the pharyngeal teeth (Fig. 5g). In horizontal sections, *enam* was more strongly expressed in the mesenchymal cells of the second pedicles than in those of the first pedicles (Fig. 6g). Transcripts for *sparc* were detected in the odontoblasts and mesenchymal cells of pedicles (Fig. 5h). *Sparc* was not detected in the mesenchymal cells around the first pedicles; however, it was expressed in mesenchymal cells around the second pedicles (Fig. 6h). Transcripts for *spp1* were weakly detected in the pulp area of the pharyngeal teeth near the pedicles and in the surrounding mesenchymal cells of the pedicles (Figs. 5i, 6i). Transcripts for *col1a1* were detected in the mesenchymal cells located on posterior side of the pedicles but not in cells within the pedicles or pharyngeal teeth (Fig. 5j). In horizontal sections, *col1a1* transcripts were detected in mesenchymal cells on the posterior side of the second pedicles but not in the first pedicles (Figs. 3g, 6j). No *bglap* transcripts were detected in the cells of pharyngeal teeth and pedicles, but transcripts were detected in osteoblasts of pharyngeal bone (Fig. 5k). Transcripts for *runx2* were detected in mesenchymal cells of the pedicles and weakly detected in the cells surrounding the pedicles, but they were not detected in pharyngeal teeth (Figs. 5l, 6k). Transcripts for *sp7* were detected in the cells on the posterior side of the second pedicles, but not in the mesenchymal cells of the pharyngeal teeth and pedicles (Figs. 5m, 6l).

**Fig. 7:**
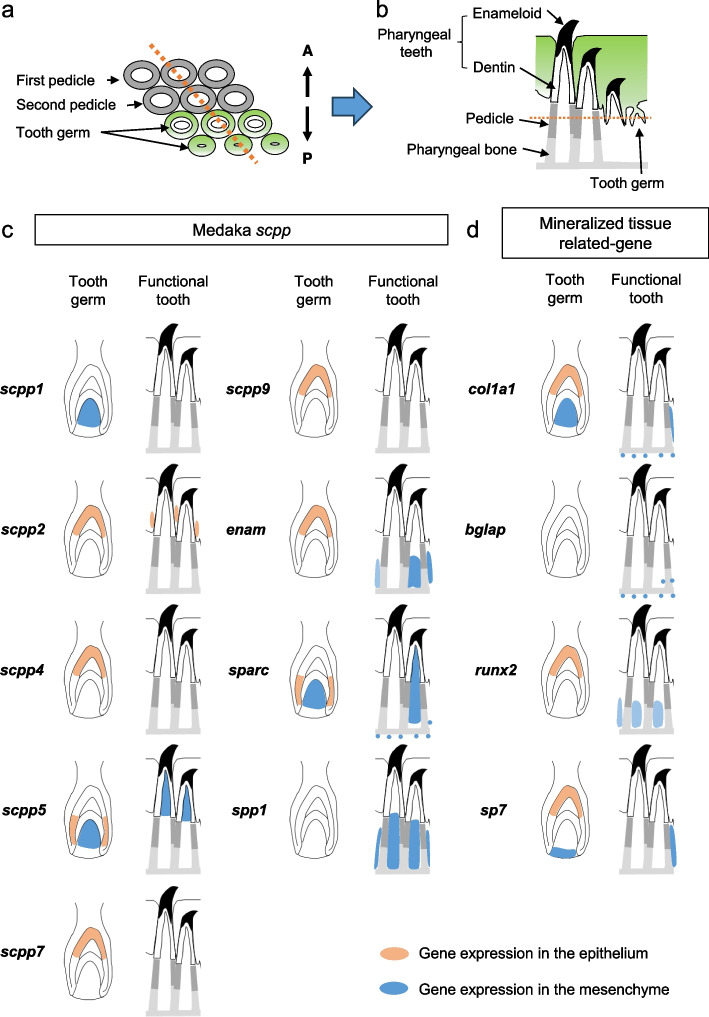
Summary of *scpp* and mineralized tissue-related genes expression patterns in the pharyngeal tissues. **a**, **b** Schematic representation of a section at the level of the pedicles under the erupted functional teeth. The schematic representation shows two pedicles and two successional tooth germs in a row. The cross-section was cut so that the pedicle and the tooth germ are continuous (orange dotted line), and the cross-section shown in the schematic is obtained on the right. **c**, **d** Schematic representation of expression patterns of *scpp* and mineralized tissue-related genes. Expression in the epithelium is shown in orange, and expression in the mesenchyme is shown in blue. The specific expression patterns of the *scpp* genes were in each stage (Fig. 7c). *Scpp1*, *scpp4*, *scpp7*, and *scpp9* were expressed in tooth germ, but not in the cells of teeth and pedicle (Fig. 7c), while transcripts of *spp1* exhibited the opposite expression pattern (Fig. 7c). The expression of *enam* was detected in the ide of tooth germ and the cells of the second pedicles (Fig. 7c). In addition, *scpp2* was consistently expressed in the epithelium (Fig. 7c). Other genes of the epithelial components that were detected in the tooth germ were not detected in the cells of functional teeth and pedicles (Fig. 7c). *Scpp5* expression was only detected in the cells that formed enameloid and dentin (Fig. 7c)

Figure 7 shows a schematic representation from the tooth germ stage to the functional teeth of medaka based on ISH data. Figure 7a was prepared with reference to the sections shown in Figs. 3h and 6. It shows two pedicles and two successional tooth germs in a row. The cross-section of same pedicles and tooth germ are shown in Fig. 7b. The areas of *scpp* and mineralized tissue-related gene expression were classified into epithelial and mesenchymal components (Fig. 7c, d). The specific expression patterns of the *scpp* genes were in each stage (Fig. 7c). In summary, *scpp1*, *scpp4*, *scpp7*, and *scpp9* were expressed in tooth germ, but not in the cells of teeth and pedicle (Fig. 7c), while transcripts of *spp1* exhibited the opposite expression pattern (Fig. 7c). The expression of *enam* was detected in the ide of tooth germ and the cells of the second pedicles (Fig. 7c). In addition, *scpp2* was consistently expressed in the epithelium (Fig. 7c). Other genes of the epithelial components that were detected in the tooth germ were not detected in the cells of functional teeth and pedicles (Fig. 7c). *Scpp5* expression was only detected in the cells that formed enameloid and dentin (Fig. 7c).

## Discussion

To investigate the genetic basis of the formation of tooth and periodontal tissues in medaka, we examined the expression of several *scpp* genes encoded in the medaka genome. The present study is the first to describe the expression patterns of *scpp* genes during pharyngeal tooth formation in medaka.

### The role of *scpp* genes in dentin formation

The expression levels of *scpp1* and *scpp5* in medaka were higher in pharyngeal tissues (Fig. 2a). Expression of *scpp1* was detected only in odontoblasts in tooth germ, and *scpp5* was expressed in both ide and odontoblasts in tooth germ (Fig. 4a, d). These tooth germ expression patterns are similar to those of fugu, zebrafish, and cichlids [7, 8, 15, 16]. Interestingly, expression of *scpp5* was detected only in odontoblasts of functional teeth, and no other *scpp* gene was expressed in these cells (Fig. 5d). In addition, seahorses, and pipefish (family *Syngnathidae*) are toothless, a phenomenon known as edentulism, and in seahorses, *scpp5* is a pseudogene [20]. Furthermore, the pharyngeal teeth in *scpp5*-deficient zebrafish exhibit a distinct phenotype characterized by fewer functional teeth [21]. In addition, the expression patterns of *scpp1* and *scpp5* were similar to those of the *dentin sialo phosphoprotein* (*Dspp*) gene in rats and mice [22, 23]. In the rat, the *Dspp* gene is expressed in odontoblasts at all stages and transiently in preameloblasts during tooth development [22]. Although the genomic position of *scpp1* in zebrafish is similar to that of *Dspp* and *dentin matrix protein 1* (*Dmp1*), the amino acid sequence of scpp1 differs from that of Dspp and Dmp1 [8]. Furthermore, no *Dspp* or *Dmp1* orthologues have been found in teleosts fish [8]. However, Mikami et al. reported that *Dspp* or *Dmp1* orthologues have been found in ray-finned fish [14]. These results suggest that both scpp1 and scpp5 in medaka play a critical role in dentin formation.

### The role of *scpp* genes in enameloid formation

Among the *scpp* genes in medaka, the expression of *scpp2*, *-4*, *-5*, *-7*, *-9*, *enam*, and *sparc* was detected in the ide (Figs. 4b, c, d, e, f, and 7). These expression patterns were consistent with those in fugu, zebrafish, and cichlid [7, 8, 15, 16]. Kawasaki et al. reported that *scpp2*, *-4*, *-5*, and *-9* play important roles during enameloid formation in fish [7, 8, 15]. The genes *scpp4*, *-5*, *-7*, and *-9* are fish specific. The *scpp2* gene encodes ODAM and is an orthologue of a gene found in mice. In mice, ODAM is expressed in the inner enamel epithelium and junctional epithelium [24, 25] where it modulates the mineralization of enamel via the regulation of matrix metalloproteinase-20 in conjunction with Runx2 [26]. The above results suggest that the expression of *scpp* genes is also involved in the formation of highly mineralized tissues such as cap enameloid (acrodin).

In fish, enameloid is usually found at the tooth cap, and it is called cap enameloid (acrodin) [11]. Moreover, in *Polypterus senegalus*, collar enamel forms at the surface of the tooth shaft [11], while only cap enameloid (acrodin) is observed in medaka. Kawasaki et al. reported that *scpp2* and *scpp9* are involved in the hyper-mineralization of collar enameloid in addition to cap enameloid (acrodin) [8]. A recent study reported that *scpp5*, *ameloblastin*, and *enam* are expressed in the ide cells adjacent to the surface of the tooth shaft in gar and zebrafish [15]. Interestingly, in the present study on medaka, no expression of *scpp5*, *scpp9*, or *enam* was detected in the cells adjacent to the surface of the tooth shaft; only *scpp2* was detected in those cells (Figs. 4b, 7). Thus, the expression of *scpp2* in medaka appears to be related to another role for ODAM in mediating active adhesion mechanisms at the junctional epithelium [27]. These data suggest that expression of *scpp2* adjacent to the surface of the tooth shaft in medaka contributes to active adhesion to the epithelial tissue adjacent to the tooth shaft rather than playing a role in hyper-mineralization.

### Other *scpp* genes

In channel catfish, *scpp7* is highly expressed in the skin [28]. In the present study, total RNA was extracted from fin ray bones without removing the epithelium, and analyses revealed that *scpp7* was strongly expressed in fin ray bone (Fig. 2a). In ISH, transcripts for *scpp7* were detected in the epithelial cells adjacent to the fin ray bone, but not in osteoblasts (Additional file 1). In addition, *scpp5*, *scpp7*, *spp1*, and *sparc* are expressed over time during scale regeneration in zebrafish [29]. These results suggest that *scpp7* expression might be critical during skin and scale formation and regeneration.

*Spp1* encodes osteopontin, a sialic acid–rich, phosphorylated, integrin-binding extracellular matrix glycoprotein that enhances osteoclast activity [30, 31]. The inhibitory effect of osteopontin on calcification depends on the degree of phosphorylation of serine [30]. In medaka, pharyngeal teeth replacement is accompanied by the remodeling of pedicles, which occurs under the first functional teeth [5]. Such dynamic tooth replacement is associated with the dynamic resorption of pedicles by the large population of TRAP-positive osteoclasts [6]. Bone matrix–resorbing osteoclasts are anchored by osteopontin bound both to the mineral of the bone matrix and to a vitronectin receptor on the osteoclast plasma membrane [32]. In addition, osteopontin is involved in cell attachment during cementogenesis in mice [10]. In the present study, strong *spp1* expression was detected in mesenchymal cells on the anterior side of pedicles (Fig. 5i). TRAP-positive cells were also observed in the anterior side of the first pedicles (Fig. 2h). In contrast, *spp1* expression was detected in mesenchymal cells on the posterior side of pedicles (Fig. 5i). These results suggest that the expression pattern of *spp1* reflects the process of tooth replacement.

*Sparc*, also known as osteonectin or basement membrane protein 40, is one of the most abundant non-collagenous proteins in bone [33]. *Sparc* is expressed in both mineralized and non-mineralized tissues [33]. Osteonectin-null mice show reduced bone formation and decreased osteoblast and osteoclast surface area and number, which leads to a decrease in bone remodeling with a negative bone balance and profound osteopenia [34]. In the present study, the expression pattern of *sparc* in the tooth germ was similar to that in fugu, zebrafish, and cichlids [7, 8, 35]. In catsharks, *sparc* is expressed in odontoblasts, and in thornback rays, it is weakly expressed in the mesenchyme cells of tooth buds [36]. These results suggest that expression of other *scpp* genes occurs during tooth development in teleosts and chondrichthyans. In the functional teeth of medaka, *sparc* was expressed continuously in the mesenchymal cells during formation of the pharyngeal teeth and pedicles (Fig. 4h). The expression pattern of *sparc* during pharyngeal teeth formation in this study was consistent with that in mice, suggesting that *sparc* has a similar function in teleosts and mammals.

### The expression patterns of mineralized tissue-related genes change during the tooth replacement process

The findings of our previous study showed that individual functional teeth and their successional teeth are organized into families [4]. The pharyngeal teeth of medaka are replaced with the next group of teeth within 4 weeks [4]. Tooth replacement in medaka involves pedicle remodeling by osteoblasts and osteoclasts [5]. In the present study, *sparc* and *col1a1*, which are osteoblast markers, were expressed in the posterior side of the first pedicles but not in the anterior side (Fig. 6h, j), and *sp7* expression was observed in the posterior side of the second pedicles (Fig. 6l). In addition, while *enam* was expressed in the second pedicle during the process of bone formation, no *enam* expression was observed in the already-formed first pedicles (Figs. 5g, 6g). In ST2 cells, exogenous *ameloblastin* and *enam* suppressed RANKL expression, which attenuated multinucleated osteoclast formation [37]. In contrast, numerous TRAP-positive cells and *spp1* expression were detected in the cells on anterior side of the first pedicles (Fig. 3h). Considered collectively, these expression patterns are consistent with the cell dynamics of pedicle remodeling during tooth replacement.

### Is the pedicle a bone-like tissue or a dentin-like tissue?

Two major points must be considered to answer this question. The first point is that *scpp* genes are specifically expressed in the cells that form dentin and pedicles. In this study, the expression of only *scpp1* and *scpp5* was detected in odontoblasts. *Scpp5* transcripts were expressed in odontoblasts during the stages from tooth germ formation to the formation of functional teeth. Previous studies have shown that *scpp5* exhibits a similar expression pattern in fugu, zebrafish, and gar [7, 8, 15]. A recent study reported that the pharyngeal teeth in *scpp5*-deficient zebrafish exhibit a distinct phenotype, particularly a decreased number of functional teeth [20], Rosa et al. reported that *scpp5* in zebrafish was expressed in the odontoblasts during the tooth germ stage, but not in the cells depositing the “bone of attachment” [17]. The expression patterns of *scpp5* in zebrafish were similar to those in medaka, implying that, *scpp5* plays a role in dentin formation in teleosts fish. In addition, the findings showed that *scpp1,* and *scpp5* were not expressed in the cells of pedicles, whereas *spp1*, e*nam*, r*unx2*, and *sp7* were expressed. Interestingly, these factors cause morphological abnormalities in the tooth roots of mammals. In mice, *Spp1* is strongly expressed by osteoblasts in alveolar bone but rarely expressed in odontoblasts [38]. In *enam*-deficient mice, excessive absorption of dentin and cementum of the tooth root was observed [39]. In *runx2*-overexpressing mice, the tooth roots form osteodentin-like structures [40]. In addition, the jaw teeth of *sp7*-mutant medaka and zebrafish fail to form pedicles [41, 42]. Micro-computed tomography analysis revealed that Osterix conditional knock-out mice have short roots and a thin dentin matrix [43]. Alterations, either through deletion or overexpression of these genes, results in abnormal tooth root morphology in mammals. These abnormalities were specifically observed in the cells responsible for forming the pedicles. Notably, similar root and pedicles phenotypes associated with *sp7* have been observed in both mice and teleosts fish.

The other point that must be considered is that the epithelial elongation of the enameloid organs terminates at a certain level during tooth formation in teleost fish such as medaka. Tooth development is controlled by interactions between the epithelium and mesenchyme. In tooth development in mammals, the cervical loop grows after the crown formation stage, becoming Hertwig’s epithelial root sheath. However, the extent to which Hertwig’s epithelial root sheath descends toward the root apex varies among species [44]. The teeth of medaka are also formed by interactions between the epithelium and mesenchyme. However, no epithelial tissue adjacent to the pedicle is observed during pedicle formation.

Rosa et al. reported that given the presence of dentinal tubule-like cell extensions and the near absence of osteocytes, the characteristics of the pedicles in zebrafish were intermediate between bone and dentin, and that this material was better termed “dentinous bone” [17]. However, the cells responsible for depositing the “bone of attachment” are more closely associated with osteoblasts, as evidenced by the presence of the osteoblast marker Zns-5 and the lack of a covering epithelium, rather than with odontoblasts [17]. While osteocytes have been confirmed in the pedicles of the jaw teeth of *Pagrus auratus* [45], medaka does not osteocytes its pedicles. This suggests that pedicles of medaka might be similar to those of zebrafish in this context.

In summary, the pedicle cannot be characterized as dentin for the following reasons: 1) the genes expressed in the teeth are not detected in the cells that form pedicles; and 2) no close spatial association with epithelial cells is detected in pedicle-forming cells. Thus, the pedicle is considered to be a bone-like tissue with characteristics that are similar to dentin.

## Conclusions

We characterized changes in the expression patterns of *scpp* genes in medaka during the formation of pharyngeal teeth and pedicles. Among the *scpp* genes, *scpp5* in medaka is considered to be a crucial gene in the tooth formation.

### Supplementary Information

**Additional file 1.** *scpp7* expression in fin ray Transcripts for *scpp7* were detected in the epithelial cells adjacent to the fin ray bone (black arrow), but not in osteoblasts in the fin ray bone (*).

**Additional file 2.** Uncropped electrophoretic gel image of figure 2c (*scpp2* and *β-actin*).

## Data Availability

The datasets generated during and/or analyzed during the current study are available from the corresponding author on reasonable request. The accession or Ensembl numbers used for data analysis were as follows. *scpp1*: XM_011475869.3, ENSORLG00000024846 *scpp2*: XM_004065876.4, ENSORLG00000006190 *scpp4*: XM_011475773.3 *scpp5*: XM_004084047.4, ENSORLG00000019321 *scpp7*: XM_020699512.2 *scpp9*: XM_004065877.3 *enamelin*: XM_011475762.3 *spp1*: XM_004086583.4, ENSORLG00000020900 *sparc*: NM_001104877.1, ENSORLG00000011369 *col1a1*: NM_001122918.2 *bglap*: NM_001201510.1 *runx2*: NM_001104850.1 *sp7*: XM_011474867.2

## Abbreviations

SCPP: Secretory calcium-binding phosphoprotein
PCR: Reverse transcription-polymerase chain reaction
qPCR: Real-time quantitative PCR
ISH: In situ Hybridization
P/Q: Pro/Gln
ODAM: Odontogenic ameloblast-associated protein
SPARCL1: Secreted protein, acidic, cysteine-rich like 1
ambn: Ameloblastin
enam: Enamelin
GDV: Genome Data Viewer
EGB: Ensembl Genome Browser
SD: Standard deviation
EDTA: Ethylenediaminetetraacetic acid
TRAP: Tartrate-resistant acid phosphatase
DIG: Digoxigenin
col1a1: Collagen 1a1
bglap: Bone gla protein
runx2: Runt-related transcription factor 2
ide: Inner dental epithelium
ode: Outer dental epithelium
Dspp: Dentin sialo phosphoprotein
Dmp1: Dentin matrix protein 1
