# Supplementary material for: Expression of secretory calcium-binding phosphoprotein (scpp) genes in medaka during the formation and replacement of pharyngeal teeth
Source: BMC Oral Health. 2023 Oct 11;23:744. doi: 10.1186/s12903-023-03498-7 (PMC10568847; doi:10.1186/s12903-023-03498-7)
Supplement: Supplementary file 1 — Additional file 1. scpp7 expression in fin ray Transcripts for scpp7 were detected in the epithelial cells adjacent to the fin ray bone (black arrow), but not in osteoblasts in the fin ray bone (*). [file 12903_2023_3498_MOESM1_ESM.pdf]

Additional file 1: *scpp7* expression in fin ray

Transcripts for *scpp7* were detected in the epithelial cells adjacent to the fin ray bone (black arrow), but not in osteoblasts in the fin ray bone (*).

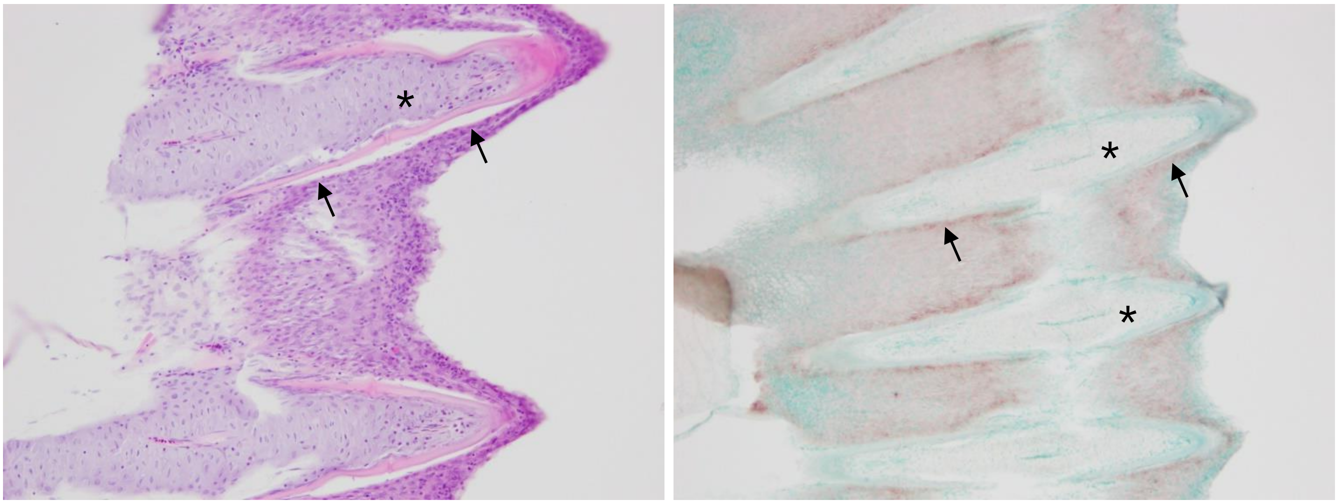

HE *scpp7*
