# Supplementary figures and images for: Expression of secretory calcium-binding phosphoprotein (scpp) genes in medaka during the formation and replacement of pharyngeal teeth
Source: BMC Oral Health. 2023 Oct 11;23:744. doi: 10.1186/s12903-023-03498-7 (PMC10568847; doi:10.1186/s12903-023-03498-7)

Additional file 2:  
uncropped electrophoretic gel image of figure 2c (*scpp2* and  $\beta$ -*actin*).

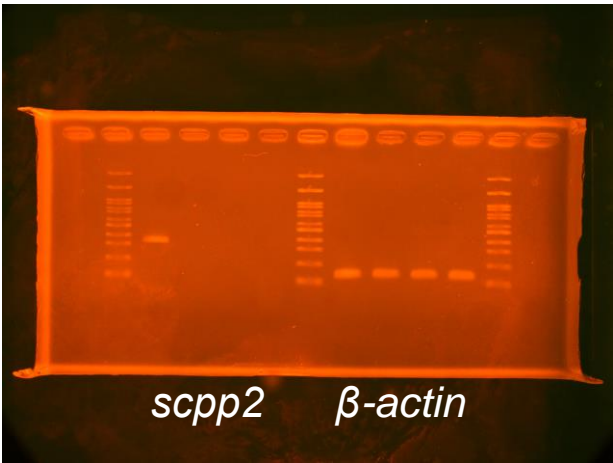

Supplement: Supplementary file 2 — Additional file 2. Uncropped electrophoretic gel image of figure 2c (scpp2 and β-actin). [file 12903_2023_3498_MOESM2_ESM.pdf]
